# *In vitro* human gut microbiota fermentation models: opportunities, challenges, and pitfalls

**DOI:** 10.20517/mrr.2022.15

**Published:** 2023-01-17

**Authors:** Julia Isenring, Lea Bircher, Annelies Geirnaert, Christophe Lacroix

**Affiliations:** Department of Health Sciences and Technology, ETH Zurich, Zürich 8092, Switzerland.

**Keywords:** Human gut microbiota, intestinal fermentation technology, *in vitro* modeling, *ex vivo *cultivation

## Abstract

The human gut microbiota (HGM) plays a pivotal role in health and disease. Consequently, nutritional and medical research focusing on HGM modulation strategies as a means of improving host health is steadily increasing. *In vitro* HGM fermentation models offer a valid complement to human and animal studies when it comes to the mechanistic exploration of novel modulation approaches and their direct effects on HGM composition and activity, while excluding interfering host effects. However, *in vitro* cultivation of HGM can be challenging due to its high oxygen sensitivity and the difficulties of accurately modeling the physio-chemical complexity of the gut environment. Despite the increased use of* in vitro *HGM models, there is no consensus about appropriate model selection and operation, sometimes leading to major deficiencies in study design and result interpretation. In this review paper, we aim to analyze crucial aspects of the application, setup and operation, data validation and result interpretation of *in vitro *HGM models. When carefully designed and implemented, *in vitro* HGM modeling is a powerful strategy for isolating and investigating biotic and abiotic factors in the HGM, as well as evaluating their effects in a controlled environment akin to the gut. Furthermore, complementary approaches combining different *in vitro* and *in vivo* models can strengthen the design and interpretation of human studies.

## INTRODUCTION

The human gut microbiota (HGM) is a complex, dynamic, and diverse ecosystem that develops in and inhabits the human intestine from birth. The HGM plays a crucial role in the host’s health status by interacting with them either directly via neural, immune, or endocrine pathways, or indirectly by providing metabolic, protective, and developmental functions^[1]^. This has led to the ongoing development of HGM modulation strategies to promote health. Simultaneously, the HGM is linked with various chronic diseases, including inflammatory bowel disease, metabolic syndrome, type 2 diabetes, colorectal cancer, metabolic disorders, and nervous-system-related diseases in a state of dysbiosis^[2-5]^. Dysbiosis is defined by Vangay *et al.* as a “loss of keystone taxa, loss of diversity, shifts in metabolic capacity, or blooms of pathogens” and most commonly occurs due to changes in the exposome^[6]^. The exposome consists of all biotic and abiotic factors to which an individual is exposed during the course of their life, such as medication, diet, and lifestyle^[7]^. However, such findings are mainly based on observational studies that fail to explain underlying complex mechanisms^[8,9]^. At the same time, it is extremely challenging to demonstrate mechanisms and causality, due to the multifactorial interplay between the HGM, the host, and its exposome, analytical restrictions, and limitations on the transmissibility of findings from animal studies to humans^[9]^.

A mechanistic understanding of how the microbiota can lead to disease and how its function can be modulated requires these complex interactions to be disentangled. A holistic approach combining *in vitro* and animal models with human studies in multiscale strategies has been promoted to decipher the role of the gut microbiota in humans and elucidate hypothesis-based mechanisms^[10-12]^. *In vitro* HGM models involving complex communities are increasingly being used in medical and nutritional research. When well designed and properly applied, they make it possible to focus entirely on microbe-microbe interactions, excluding host factors, and are highly suitable for assessing the impact of abiotic or biotic factors from the exposome. Furthermore, *in vitro* HGM fermentation models can significantly reduce the use of animal testing, which is desirable in light of societal and ethical considerations, and the generated data can be used to optimize *in vivo* research protocols. For example, evaluating the effectiveness of dietary compounds or drugs in *in vitro *HGM models enables quality adaptation and improvement prior to *in vivo *testing^[13,14]^.

Different *in vitro* HGM model configurations have been employed, from simple batch incubation in anaerobic, here referred to as conditions without an inflow of oxygen, to fully controlled, continuously operated, and complex multistage models. It is crucial to choose model(s) that are appropriate for answering a clearly defined research question. Nevertheless, there is no consensus on the choice of model, experimental setup, indicators for successful cultivation, how closely the host microbiota can be mimicked, or the conclusions that can be appropriately drawn from data obtained through* in vitro *HGM studies. This can lead to spurious data reports characterized by an imbalance in microbiota composition and activity, inappropriate extrapolation of results, and an inability to compare different *in vitro* studies. For this reason, we focus here on drawing attention to the most critical points for the proper application, setup, and result interpretation of *in vitro *HGM models.

## RELEVANT CHARACTERISTICS OF HGM AND ITS ENVIRONMENT FOR CULTIVATION ASSAYS

Conditions in the small and large intestines differ greatly. The small intestine, where most nutrients are degraded and absorbed by the host, is characterized by sharp gradients of biotic and abiotic factors that strongly limit the application of relevant models. It is characterized by heterogenous microenvironments, steep longitudinal gradients in nutrients (temporal and spatial), pH, and oxygen, and a short transit time of 2-5 h^[15-18]^. Thus, the microbial diversity and load is rather low compared to the colon, with 10^4^ to 10^7^ bacteria/g^[16]^. Further, obtaining a representative sample of small intestine content is almost impossible. Ileostomy samples are sometimes used as proxies, but most people with a stoma are not categorized as healthy individuals and the presence of a stoma can itself change the local environmental conditions (e.g., contamination with air). Thus, there is a lack of knowledge regarding the small intestinal HGM and the biotic and abiotic gradients of the small intestine, as well as representative reference data that would validate a given model. Consequently, most small intestine models are developed for the digestion and absorption of food but lack a microbiota component. Some recent attempts have been made to model fermentation or specific conditions in the small intestine^[19-21]^. However, important shortcuts still remain: the use of fecal, ileostomy, or single-strain inoculum, operation conditions not well representing the *in vivo* gradients and medium formulated to mimic the chyme entering the colon^[22,23]^. A batch model of complete ileostomy fluid as a source of microbiota and nutrients could be an interesting approach for small intestine fermentation.

The colonic HGM is a highly diverse mixture of microbes, mainly composed of anaerobic bacteria but also containing archaea, fungi, protozoa, bacteriophages, and viruses^[24-28]^. Due to its great abundance and functional importance, most research focuses primarily on the bacterial fraction of the HGM^[29]^. It has been estimated that the human colon is densely inhabited by approximately 100 trillion bacteria cells, classified into hundreds of different species and distributed among the five dominant phyla: Firmicutes (new name: Bacillota), Bacteroidetes (Bacteroidota), Actinobacteria (Actinomycetota), Verrucomicrobia (Verrucomicrobiota), and Proteobacteria (Pseudomonadota)^[30-32]^. While the occurrence of the different phyla is highly conserved among subjects, there is large inter-individual variability in terms of species- and strain-level composition^[33]^. In contrast, metabolic pathways reconstructed from metagenomic data were shown to be stable among individuals, despite variations in community structure^[34,35]^. Members of the HGM actively shape the community composition through a dynamic network built on positive microbe-microbe interactions (e.g., the exchange of nutrients) as well as negative ones (e.g., the excretion of toxins)^[36]^. So far, only 30% of the species diversity present in the HGM has been covered by cultured representatives^[37]^.

While the isolation of gut microbes has proven difficult and laborious, mainly due to the unknown nutritional and physicochemical requirements of individual species, it is considered crucial for an in-depth functional understanding of the HGM^[31,38]^. This underlines the importance of *in vitro* HGM models that enable the growth of such fastidious and as yet uncultured microbes within a complex community via cross-feeding mechanisms between community members, by supplying the necessary growth factors in the fermentation medium^[39]^. To maintain the composition and activity profile of an HGM *in vitro*, culture conditions need to be carefully set to reflect the condition prevailing in the anaerobic gut environment of the human donor. However, chemical and physiological parameters, including pH, oxygen pressure, redox potential, transit time of the intestinal content, nutrient supply, and host secretions, vary between different intestinal sections and consequently shape the composition and activity of the resident communities according to axial and vertical gradients along the gastrointestinal tract^[40]^. For example, partial oxygen pressure and thus the redox potential, decreases along the radial axis from the mucosa to the lumen, leading to higher proportions of facultative anaerobes from the phyla Proteobacteria and Actinobacteria in samples from rectal mucosa than in feces^[41,42]^. Therefore, selection of the HGM *in vitro* model parameters depends heavily on the anatomical region intended to be mimicked in the experimental setup, as gradients cannot be achieved in well-mixed models. Furthermore, compromises must be made concerning distinct host-specific features, such as the absorption of microbial products, host enzymes, and the secretion of antibodies that are difficult to model *in vitro*. In contrast, chemical and physical parameters, including temperature, pH, redox potential, mixing, and transit time (retention time), are relatively easy to translate to *in vitro* systems, but vary among individuals. Therefore, representative median conditions for the target population must be carefully selected according to well-identified criteria, such as age, diet, and general health status.

The metabolic activity of the modeled HGM can be assessed directly by targeted metabolite analysis or global methods, such as metatranscriptomics (the entirety of expressed genes) or metaproteomics (the entirety of proteins). An overview of the role of omics approaches in HGM research can be found elsewhere^[43]^. In practice, HGM activity is mostly monitored by chromatography methods. One of the main functions of the HGM is to break down undigested carbohydrates, proteins, and peptides to form the main short-chain fatty acids (SCFA): acetate, propionate, and butyrate^[44]^. Measuring these metabolites is a good and commonly applied functional readout of HGM *in vitro* models. In addition, intermediate metabolites (e.g., succinate, lactate, and formate), branched-chain fatty acids (BCFAs) from proteolytic fermentation and the microbial gasses H_2_, CO_2_ and CH_4_, and any measurable metabolic product can be included in the analysis to obtain a more complete description of the metabolic activity of the modeled HGM^[45,46]^. HGM composition can be assessed in a targeted manner using 16S rRNA gene amplicon sequencing or quantitative real-time PCR (qPCR). 16S rRNA gene amplicon sequencing identifies bacteria taxa down to the genus level and correspondingly generates relative abundance data^[47]^. In parallel, qPCR or flow cytometry enables the enumeration of the total bacterial load of the HGM samples and the quantification of low abundant taxa that are below detection in 16S rRNA gene amplicon sequencing^[48]^. In contrast to the targeted methods, shotgun metagenomic sequencing (MGS) extends the phylogenetic resolution to the species or strain level, provides additional functional information, and can cover as yet uncultured bacterial genomes^[49]^. Usually, shotgun sequencing is more expensive than 16S rRNA gene amplicon sequencing, largely due to the higher sequencing depth needed. However, recent research suggests using shallow shotgun MGS as a similarly priced alternative to 16S rRNA gene amplicon sequencing, while identifying further species and providing similar information to deep shotgun MGS^[50,51]^.

In conclusion, the strength and validity of HGM models is amplified by the rational selection of operating physico-chemical parameters, combined with sample accessibility and rapidly evolving analytical methods.

## THE CHOICE AND PROCESSING OF STARTING MICROBIOTA MATERIALS IMPACTS THE QUALITY OF MODEL DATA

### Source of microbiota

Due to widespread availability and easy collection of feces, liquid suspensions of fecal microbiota are most commonly used as inoculum for HGM models. It may be argued that a fecal microbiota that has undergone complete gastrointestinal transit is compositionally different from the communities that are generally targeted by intestinal fermentation models, e.g., the proximal colon section, where most microbial growth and metabolism occur^[52,53]^. Thus, fecal microbiota is a proxy for the intestinal microbial community, and changes in composition and activity are expected in *in vitro* fermentations^[54]^. However, assuming that fecal microbiota harbors most of the species present in the gut in a viable state - albeit at possibly different rations - the composition of the modeled gut microbiota can be driven to the composition of the proximal colon by carefully controlling the initial fermentation phase and the operating conditions of a continuous model. Another approach is to use artificial HGM produced by intestinal fermentation technology from a donor inoculum. After carefully selecting the model and controlling conditions (presented in the section “Selection of model type and operation according to the research question”), a standardized and characterized HGM community can be obtained in large quantities through *in vitro *fermentation technology^[55,56]^. However, the quality depends on the modeling capacity and there is less diversity than in a fecal sample.

### Fresh microbiota as the gold standard

HGM models are strongly impacted by the quality of the inoculum microbiota, including the viability and fitness of the different species. Some researchers have suggested using cryopreserved fecal microbiota as an alternative to fresh feces, in order to extend the time frame and increase the number of studies that can be performed with the same microbiota^[57]^. However, freezing can induce osmotic and mechanical stresses on bacterial cells that can result in disruption of the cell membranes and ultimately cell death. Differences in susceptibility to freezing-induced stresses might result in shifts in microbial composition and activity, with resistant bacteria groups favored upon reactivation, while the growth of sensitive taxa is impaired^[58]^. This can add significant bias to the model and treatment effects. Using HGM cultivated in the PolyFermS model, we showed that functions that are highly redundant in HGM are better maintained upon snap-freezing and storage than functions shared only between a few taxa^[54]^. Furthermore, bacterial viability in fecal samples was lower upon oxygen exposure (19%) and freeze-thawing (23%) compared to fresh samples (50%)^[59]^. We recently demonstrated that cooling and air protection during the transportation of Kenyan infant microbiota makes it possible to extend the time frame between sampling and reinoculation of HGM PolyFermS models to 30 h, without major loss of diversity between the native and artificially produced microbiota (unpublished data). Nevertheless, if fresh inocula cannot be used, fecal samples should at least be treated with a cryoprotective solution, e.g., a protective buffer containing 10%-15% glycerol^[54,57,60]^, which is ideally removed prior to inoculation, to prevent cryoprotectant from impacting bacterial growth. Whenever possible, however, HGM model inoculation should be done using fresh microbiota.

### Pooling microbiota may not be optimal

Depending on the HGM model and experimental testing volume, a high inoculation might be required, necessitating significant amounts of starting fecal inoculum material, which can be difficult or even impossible to collect from a single donor (e.g., from infants). Pooling fecal microbiota from different donors has therefore been done to increase the amount of fecal inoculum, possibly combined with frozen storage for repetitive use or to obtain a “more standardized” fecal inoculum^[57]^. However, research has shown that microbiota interventions, such as probiotics or prebiotics, do not have uniform effects across different subjects, but rather their outcome depends on the individual’s baseline microbiota, as shown both *in vivo*^[61-63]^ and *in vitro*^[64-68]^. By pooling fecal microbiota, interindividual differences are completely removed and an “artificial” community with unpredictable competition and balance among taxa is created. In contrast to the recent suggestion that microbiota should be pooled for batch fermentations^[69]^, we recommend the use of individual, well-protected, and fresh fecal microbiota as an inoculum for *in vitro *HGM experiments to prevent unforeseeable biases.

### Selection of model type and operation according to the research question

*In vitro *HGM models range from deep-well plates to fully controlled bioreactors. They can be operated in batch, semi-continuous, and continuous modes and set up as single- or multi-stage models. They have been reviewed previously^[70-72]^.

Upon inoculation, the gut microbial community will adapt from its natural environment, i.e., the donor intestine, to its new environment created *in vitro*. The chosen HGM model strategy will therefore significantly determine this community adaptation and the type of readouts and conclusions one can draw. In particular, the initial conditions of the fermentation must be carefully selected and adjusted to prevent the overgrowth of fast-growing bacterial populations at the expense of more sensitive species, resulting in important deviations in the community balance from the target model community. Next, the cultivation parameters in HGM models should be carefully selected to mimic the gut physico-chemical conditions of the target population group, which may be challenging due to limited or missing *in vivo *data. The nutritive medium used to mimic the chyme entering the colon of the modeled host is often overlooked, but has a major impact on *in vitro* community establishment. The colon microbiota cultivation media should be adapted according to the diet of the target population, following a clear rationale. For example, media have been developed to mimic the diet of formula-fed infants^[73-75]^ and African infants living in low-income countries. In contrast, when assessing the infant gut microbiota at weaning age, cultivation media are supplemented with dietary compounds from their first solid food^[76,77]^. Finally, it is necessary to carefully evaluate whether a particular test compound should undergo suitable pre-treatments mimicking upper gastrointestinal tract digestion (e.g., INFOGEST) prior to colon model incubation^[69,78]^. Several studies have applied undigested foods or components (e.g., protein, chocolate, ice cream, *etc.*) that are known to be degraded and absorbed mainly in the upper digestive tract directly to HGM models. The external validity of such experiments is highly doubtful and the data obtained may lead to completely wrong conclusions regarding the presumed effects on the HGM *in vivo*.

**Figure 1:**
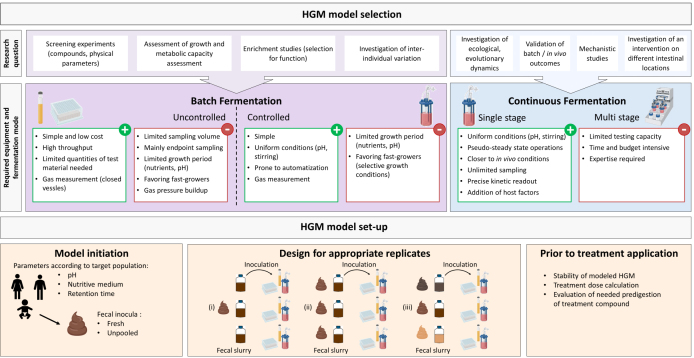
Necessary considerations for the setup of a well-designed *in vitro* human gut microbiota (HGM) model study. Based on the research question, the most suitable HGM model should be selected and setup with operational parameters that correspond with the features of the target population. Carefully determining the features of the treatment and the organization of replicates will add validity to generated data and facilitate outcome interpretation.

One key consideration is that the model type and its operation must be carefully chosen in light of well-formulated research questions, as there is no universal fit [Figure 1].

#### Batch models

Batch HGM experiments consist of the incubation of viable microbiota under strictly anaerobic conditions in a selected nutritional medium to mimic the colon nutrients for a short, fixed period of time. Batch HGM models can vary in terms of scale [multiple-well (0.5-2 mL), closed tubes or flasks (10-50 mL), bioreactor], control (pH, anaerobiosis), inoculation level, nutritional medium, and pre-treatment strategy (predigested or native components). A further important model characteristic is found at the level of feedback control during incubation. Controlled fermentations (e.g., in bioreactors) may be more complex, but allow for full and accurate control of environmental conditions, including mixing, uniform temperature, and pH (e.g., by addition of a base), anaerobiosis (gas flushing), and accurate monitoring of the kinetics and redox potential.

Batch experiments are most suitable for screening microbial growth or bioconversion capacity under the selected treatment conditions, in addition to being used for enrichment steps and as a proxy for distal colon fermentation^[79-81]^. The multiple-well batch setup in an anaerobic chamber is simple, accessible, and appropriate for high-throughput experiments with different donor microbiota or treatment conditions in parallel^[82,83]^. Nevertheless, handling small volumes necessitates strict control of anaerobiosis and cross-contamination. In contrast, the closed tubes approach can be used to monitor treatment impact on microbial kinetics, gas production, and gas composition, as demonstrated for different dietary fibers, and can be applied outside of an anaerobic chamber when using strict anaerobic Hungate technology^[84,85]^. It is important to note that sampling of a closed-batch system over time can disturb fermentation conditions, and therefore it is necessary to include multiple replicate tubes for sampling over time.

The inoculation ratio (HGM suspension to total volume) should be selected based on the research question. A low inoculation ratio (0.1%-1%) is best used to monitor the growth capacity of the total microbiota and its specific members under the applied incubation conditions. In contrast, a high inoculation ratio (10%-80%) makes it possible to measure the microbial bioconversion of a selected substrate under limited growth and can serve as readout of the metabolic capacity of the microbiota^[85,86]^. The nutritional media used for batch fermentation range from minimal to rich. Rich nutritional media mimicking the gut chyme (e.g., Macfarlane)^[87]^ supports the growth of most microbial taxa, while minimal media containing minerals and buffer solution are often used to track the microbial metabolism of a particular compound with limited growth.

Batch fermentations are most frequently performed due to their easy operation, simple setup, and high throughput. However, batch experiments are limited by the depletion of nutrients, decrease in pH, and the accumulation of fermentation- and pH-dependent growth-inhibiting metabolites over time, which restricts the incubation time to 24-48 h^[60]^. Hence, while batch experiments are very useful for testing different biotic and abiotic conditions on microbiota activity, they are less suitable for mimicking long-term HGM dynamics upon treatment compared to semi-continuous and continuous HGM models.

#### Chemostat models

Chemostat HGM models are best used to investigate community dynamics, cross-feeding mechanisms, and treatment responses on the gut microbiota under the physiological conditions of the human gut. These models consist of bioreactors, in which fresh nutritive medium is supplied in semi-continuous (e.g., SHIME^[88]^) or continuous (e.g., Gibson model^[89]^, PolyFermS^[90]^) mode and fermented effluent containing metabolic end products and microbes is removed at the same rate to maintain a constant volume. One exception here is the TIM-2 model, in which metabolite absorption is mimicked via an integrated dialysis system^[91]^. However, the scale and complexity of the model restricts the operation time to three days, strongly limiting the stabilization of the modeled HGM. Single-stage chemostat HGM models fed with a medium that mimics the chyme entering the colon are most suitable for reproducing the conditions of the proximal colon region and can be operated over time periods ranging from two weeks to several months, depending on the model. For the comparative testing of treatments on the same microbiota, several reactors can be inoculated with the same fecal slurry. Alternatively, the “artificial” HGM produced in a well-controlled continuous reactor can serve as continuous inoculum for second-stage bioreactors mounted in parallel. This design, demonstrated with the PolyFermS model, makes it possible to simultaneously test multiple treatments with the same microbiota generated in the immobilized cell inoculum reactor, as well as to attain unparalleled accuracy in the comparison of effects on microbiota composition and activity^[67,92,93]^.

Multiple-stage chemostat HGM models, such as the pioneering three-stage Gibson model, have been used to mimic the planktonic microbiota of different colon regions (ascending, transversal, and descending colon) and have also been combined with parallel configurations^[89,94]^. Through the rational selection of conditions, these models make it possible to monitor temporal and regional colon microbial dynamics. Certain chemostat models mimic both the planktonic (gut lumen) and sessile (mucosa-associated) gut microbial lifestyle using a range of strategies: e.g., by providing discrete mucin agar-coated carriers (M-SHIME)^[95]^ or mucin-alginate beads (M-ARCOL)^[96]^; by using mucin-primed packed-column biofilm reactors^[97]^; or through microbial immobilization in porous gellan-xanthan beads with mucin provided in a liquid cultivation medium (PolyFermS)^[90]^. The former approaches have the disadvantage that mucin is consumed, thus necessitating the regular renewal of mucin carriers or beads during chemostat operation, which requires the reactor to be opened and is labor-intensive. The advantage of the PolyFermS immobilized microbiota is that the gel bead composition and integrity is stable over months of continuous cultivation. Moreover, from the inoculum community, slow-growing or biofilm-associated taxa can be maintained *in vitro*, and the model exhibits remarkable stability during operation for up to 4 months^[66,98]^.

HGM stability in chemostat models is impacted by many factors including the level of control of the applied conditions, such as anaerobiosis, pH, mixing efficiency, pumping, and dilution rate. Hence, the stabilization period after inoculation with fecal microbiota differs between chemostat models, but typically extends over 7 to 14 days. Optimally, HGM stability would be assessed based on community composition. This is impractical, however, due to the lag time in the result of 16S rRNA gene amplicon sequencing during chemostat operation. Thus, the production of SCFAs is accepted as a quick and convenient read-out for community stability during operation. At present, the stability of a model is normally indicated by a day-to-day variation threshold, usually set at 10%^[67]^ or 20%^[99]^ to account for natural variation in the control units and the accuracy of the analyses. However, it is the case with all models that treatments should be applied to stable communities to distinguish as well as possible between treatment effects and naturally occurring community fluctuations.

The setup and operation of chemostat HGM models requires infrastructure and expert knowledge in fermentation technology, is labor-intensive, low-throughput, and costly. Nonetheless, it provides key information about the establishment, composition, and metabolic balances of HGM in response to many possible conditions and treatments.

After selection of a suitable HGM model, the experimental setup is very important to maximize the internal and external validity of the measured responses. This includes verifying that the modeled HGM is representative of the donor’s HGM, accounting for necessary repetitions and broadening the explanatory power of data by testing the research question using different donor microbiota.

### Validity of data generated in modeled HGM

#### Critically assessing the modeled HGM composition and activity is key to validity

Prior to result interpretation, it is crucial to examine the ability of the HGM model to reproduce a bacterial community akin to the chosen intestinal compartment and to critically assess the relevance of the composition and activity of the generated *in vitro* community in comparison with the target population and donor microbiota. The ratio of the main SCFA (acetate, propionate, and butyrate) should be comparable to the fecal ratios. In contrast, the measured absolute SCFA concentrations are always higher than in feces due to the lack of absorption by the intestinal epithelium^[90]^.

Another main factor is the density of the bacterial population reached in the fermented medium, either at the end of batch culture or after the stabilization of continuous HGM models. *In vitro* studies reporting low bacterial density are likely less relevant for mimicking microbial interactions, including competition, antagonism, commensalism, and cross-feeding mechanisms present in the gut microbiota. Typical bacterial densities of around 10^10^ cells per mL medium are indicative of model quality, given that water reabsorption in the distal colon concentrates bacterial cells approximately tenfold^[10,93]^. On the composition level, comparing the diversity of fecal and model microbiota is insufficient to demonstrate model validity. The dominant and subdominant microbial genera measured *in vitro* should also be comparable to those in the inoculum or simulated gut region. This comparison is mostly made at the genus level due to the resolution limit of 16S rRNA gene sequencing, but it can be more accurately carried out at strain level using shotgun metagenomics. Blooms in Bacteroidetes and *Enterobacteriaceae* genera are frequently observed *in vitro*, especially in proximal colon models, which may reflect the growth advantage conferred on them by the continuous supply of nutritive media containing complex glycans and glycoproteins, due to their high glycan-degrading capacity^[100,101]^. An increase in enterobacteria or lactobacilli may indicate oxygen stress during the model’s setup, whereas a loss of *Ruminococcaceae* taxa may be due to a lack of B vitamins in the nutritive medium, as autotrophy was demonstrated for several butyrate-producing *Ruminococcaceae* taxa^[102,103]^. Missing nutrients due to unknown or unavailable growth factors (e.g., specific mother-milk oligosaccharides) may explain a large fraction of the loss of specific taxa, e.g., *Bifidobacterium *sp. in infant HGM models^[104]^, which may limit the validity of the model data. Finally, the modeled HGM should represent the functionality present in the fecal sample. It is frequently assessed using PICRUSt2, although predictability is limited due to dependence on reference databases and the taxonomic limit of 16S rRNA gene amplicon sequencing^[105,106]^. Functionality can be determined more accurately based on the HGM gene pool using shotgun MGS^[107]^.

#### Include appropriate replicates in the experimental design

To boost confidence in an observed treatment effect, it is pivotal that the effect is reproducible when the treatment is applied to the same HGM, ideally by preparing several fecal slurries from the same fecal sample to inoculate the HGM model or by using independently collected fecal samples from the same donor [Figure 1]. Independent repeated sampling should be done over short time periods, since the microbiota sample may change significantly due to alterations in diet, medication, or lifestyle. It has been shown that careful planning of independent sampling within 6 months can lead to good experimental reproducibility^[66]^. Meanwhile, most HGM chemostat studies lack treatment repetition on the same microbiota when parallel reactors are used to compare different treatments. Some studies apply consecutive treatments to the same bioreactor after a washout period. However, a bias will be introduced, since history effects are often seen when the HGM is exposed to a first set of treatments. The PolyFermS model makes it possible to completely remove the effect of the previous treatment, by disconnecting, cleaning, and reconnecting to the inoculum reactor containing immobilized microbiota and restabilizing second-stage reactors under control conditions before carrying out a repetition or beginning a new treatment period.

#### Universality of a treatment effect

As we have seen, a treatment effect may depend significantly on the donor gut microbiota. It might therefore be advantageous to apply the same treatment to microbiota with distinct compositional profiles [Figure 1]. Selecting different donor microbiota to compare their response to the same treatment increases the translation power of well-designed and operated models. Currently, data obtained from replicates with distinct microbiota are either averaged for the purposes of data analysis or presented separately. We strongly recommend against averaging data from several different gut microbiota profiles prior to analysis, because microbiota-specific treatment responses will be lost. Instead, treatment effects should be evaluated for each microbiota separately and then qualitatively compared to each other. 

In summary, replicates using both the same and distinct microbiota should generally be done in HGM models to increase confidence and the validity of the results. Due to the low throughput of continuous systems, we suggest performing the experiment at least twice with the same gut microbiota and with a minimum of two distinct microbiota. This use of different microbiota not only enhances the validity of the results obtained, but may provide insights into shared features among individuals with similar treatment responses that could facilitate the development of personalized microbiota modulation strategies.

## CONCLUSION

**Figure 2:**
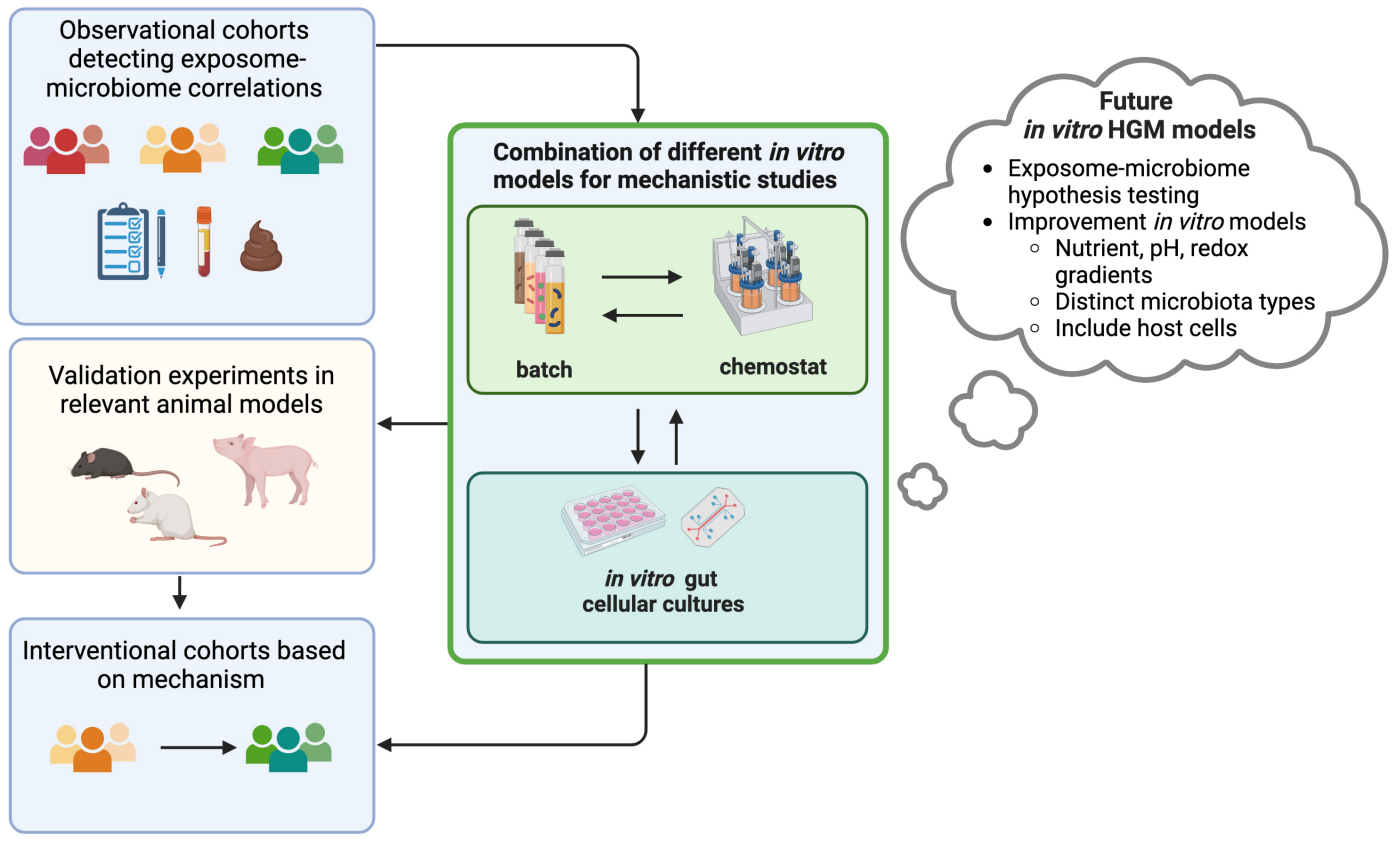
Role of *in vitro* human gut microbiota models in gut microbiome research (figure created with BioRender).

*In vitro* HGM models are excellent tools for HGM research [Figure 2], but we should be aware of certain pitfalls and recognize the limits of models which are only representations of reality. The incorrect application or operation of the *in vitro* HGM model, together with the poor interpretation of or wrong extrapolation from the data obtained, presents a risk for this growing research field, potentially leading to unrealistic expectations concerning *in vitro* HGM models, incorrect associations between the HGM and health, and unfounded speculation about the treatments for HGM-related diseases. The points and suggestions made in this paper therefore serve as guidelines for both new users and other interested parties.

We are convinced that there are several opportunities for the application and future development of *in vitro *HGM models [Figure 2]. First of all, they can be used to test and validate hypotheses made on the basis of exposome-microbiome correlations detected in large human cohort studies. Full control over abiotic factors during *in vitro* long-term HGM cultivation also makes possible the testing and validation of microbial ecology theory using a complex but also more diverse community, compared to synthetic consortia. Second, growing knowledge about the physiology of the gut and factors determining the gut microbiome in health and disease can also be integrated, in order to improve* in vitro* HGM configurations, operations, and nutrient parameters. For example, the development of *in vitro* HGM models for different microbial community states in health (different enterotypes) or disease. Third, the combination of different types of* in vitro *HGM models enables us to expand the conclusions drawn from the data obtained. For example, batch HGM experiments to screen treatments or different donor microbiota can be complemented with chemostat experiments to further investigate the impact of a particular treatment on the community structure of a selected microbiota type. *In vitro*-produced proximal colon microbiota from chemostat models can also serve as a stable and readily available inoculum for batch experiments^[55]^. Finally, a combination of *in vitro* HGM with *in vitro* host cell models can give insights into host-microbiota interactions while avoiding animal testing.

Despite the multiple opportunities for *in vitro *HGM models, certain challenges, such as the miniaturization and increased throughput of chemostat models, remain. Current chemostat setups also do not allow for the accurate application of nutrient, pH, and redox gradients as they occur in the human gut. Furthermore, an approach combining HGM fermentation with host cellular models remains under-researched, presenting specific challenges in regard to controlled gradients and maintaining meaningful host cell and bacterial viability.

## DECLARATIONS

### Copyright

© The Author(s) 2023.
